# CEFEPIME/ENMETAZOBACTAM: Physicochemical Stability of a Novel β-Lactam/β-Lactamase Inhibitor Combination in Syringes and Elastomeric Devices

**DOI:** 10.3390/antibiotics15020114

**Published:** 2026-01-23

**Authors:** Akim Chayem, Juan Quevedo, Sandrine Cure, Noëlle Jemmely, Béatrice Demore, Beatriz Esteban-Cartelle, Brayan J. Anaya, Gabriel A. Peñalver, Dolores R. Serrano, Elise D’Huart

**Affiliations:** 1 Pharmacy Department, University Hospital, Allée du Morvan, 54511 Vandœuvre-lès-Nancy, France; b.demore@chru-nancy.fr; 2 Advanz Pharma UK, London EC2M 1QS, UK; juan.quevedo@advanzpharma.com (J.Q.); sandrine.cure@advanzpharma.com (S.C.); noelle.jemmely@advanzpharma.com (N.J.); 3 INSPIIRE, Inserm, Université de Lorraine, 54000 Nancy, France; 4 Pharmacy Department, Ramón y Cajal Hospital, IRYCIS, 28034 Madrid, Spain; 5 Department of Pharmaceutics and Food Technology, School of Pharmacy, Complutense University of Madrid, 28040 Madrid, Spain; branaya@ucm.es (B.J.A.); drserran@ucm.es (D.R.S.); 6 Unidad de Espectrometría de Masas, CAI Técnicas Químicas, Facultad de Ciencias Químicas, Universidad Complutense de Madrid, 28040 Madrid, Spain; gabpenal@ucm.es; 7 Instituto Universitario de Farmacia Industrial, 28040 Madrid, Spain; 8 Infostab, 54170 Heillecourt, France

**Keywords:** prolonged infusion, stability, OPAT, ICU, antibiotic, cefepime, enmetazobactam

## Abstract

**Background:** Cefepime/enmetazobactam (FEP/META) is a novel fixed-dose β-lactam/β-lactamase inhibitor combination. The objective was to study the physicochemical stability of the approved daily dose in polypropylene syringes and elastomeric devices over a 24 or 72 h period to understand the feasibility of using FEP/META in prolonged infusions and its use for outpatient parenteral antibiotic therapy (OPAT). **Methods:** Solutions of FEP/META were prepared in 0.9% NaCl or 5% dextrose (D5W) and stored in syringes (6 g/1.5 g/48 mL) or silicone and polyisoprene elastomeric devices (EDs) at 6 g/1.5 g/120 mL and 6 g/1.5 g/240 mL: syringes were tested at 22–25 °C over a 24 h period, polyisoprene EDs at 2–8 °C over 72 h period, and silicone and polyisoprene EDs at 32 °C over a 24 h period. The solution was considered stable if it retained more than 90% of its initial concentration (Ci), no pH variation (±1 unit), no significant visual change, and with compliant subvisual examination. Liquid Chromatography–Electrospray Ionization–Quadrupole Time-of-Flight–Mass Spectrometry was utilized to identify intermediate degradation products. **Results:** At the daily dose, FEP/META retained >90% of its Ci up to 12 h in 0.9% NaCl and 24 h in D5W when stored in syringes. In silicone ED, stability was enhanced up to 24 h in D5W at all concentrations. The solution was chemically stable for 24 h when stored in polyisoprene ED in 0.9% NaCl at 2–8 °C. **Conclusions:** FEP/META combination showed prolonged stability with physicochemical integrity up to 12–24 h in all containers and conditions. It appears to be stable for prolonged infusions and for OPAT.

## 1. Introduction

Optimizing antibiotic exposure in critically ill patients has become a central objective of modern anti-infective therapy. For β-lactam antibiotics, whose efficacy is primarily driven by the time that free drug concentrations remain above the minimum inhibitory concentration (fT > MIC), prolonged or continuous infusion strategies have gained growing clinical acceptance. These approaches improve pharmacokinetic/pharmacodynamic (PK/PD) target attainment in patients with high variability of drug disposition, such as those in intensive care units (ICUs). Recent reviews and consensus guidelines have highlighted that extended or continuous infusions can increase fT > MIC, enhance bacterial killing, and, in selected patient populations, may improve clinical outcomes, including higher cure rates and reduced ICU stay durations [1,2].

Clinical evidence supporting prolonged infusion of β-lactams has expanded considerably in recent years. Meta-analyses and randomized trials suggest potential benefits, particularly in patients with severe infections or pathogens with higher MICs, although results remain heterogeneous across clinical endpoints. Nevertheless, the practical and pharmacodynamic rationale for extended infusions is widely accepted as a cornerstone of precision dosing in critically ill patients [1,3].

Beyond the hospital setting, the growth of Outpatient Parenteral Antimicrobial Therapy (OPAT) programs and the use of portable elastomeric infusion pumps have made extended β-lactam infusion feasible in home care environments. For a β-lactam/β-lactamase inhibitor (BL/BLI) combination to be suitable for OPAT, it must not only exhibit an appropriate PK/PD profile but also demonstrate adequate physicochemical stability and device compatibility in the intended diluents, containers, and temperature conditions of clinical use. Recent systematic reviews of antibiotic stability in elastomeric devices have shown that, while some β-lactams (e.g., meropenem) present stability limitations, others can safely sustain 24 h continuous infusion when stored and handled under validated, pharmacopeia-compliant conditions [4,5,6,7,8,9,10].

Within this evolving therapeutic framework, new BL/BLI combinations have attracted increasing attention due to their ability to restore β-lactam activity against extended-spectrum β-lactamase (ESBL) and other resistant pathogens. Adopting prolonged infusion regimens for these combinations requires dual validation: (i) an improved pharmacokinetic and pharmacodynamic profile and (ii) demonstration of physicochemical stability under real-world infusion settings. Consequently, before describing the specific characteristics of cefepime/enmetazobactam (FEP/META), it is essential to contextualize its role within this broader dosing optimization paradigm for BL/BLI agents and OPAT practice and to emphasize the importance of stability studies supporting its safe implementation in elastomeric infusion systems.

Enmetazobactam (META) is the first β-lactamase inhibitor approved in combination with cefepime (FEP) with a completed clinical trial [11]. The newly FEP/META, BL/BLI combination, was authorized by both the U.S. Food and Drug Administration (FDA) and the European Medicines Agency (EMA) for the treatment of complicated urinary tract infections (cUTIs), including pyelonephritis. In the European Union, the approved indications also include hospital-acquired pneumonia (HAP), including ventilator-associated pneumonia (VAP), as well as bacteraemia associated with these infections in adults [12,13].

**Figure 1:**
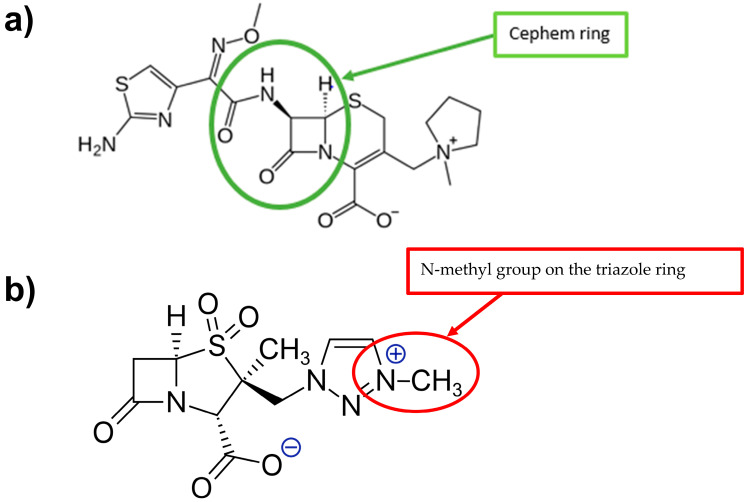
Chemical structure of FEP (**a**) and META (**b**).

META is a penicillanic acid sulfone β-lactamase inhibitor that is structurally related to tazobactam, differing primarily by the presence of a strategically placed N-methyl group on the triazole ring, which confers a net neutral (zwitterionic) charge and enhances bacterial penetration (Figure 1).

FEP carries a 2-amino-thiazolyl acetamido group with an α-oxyimino substitution at the 7-position of the cephem ring, which boosts resistance to enzymatic degradation. Additionally, the zwitterionic nature of FEP via its quaternized N-methyl-pyrrolidine moiety at the 3-position enables rapid penetration through Gram-negative outer membrane porins (Figure 1).

While META binds to β-lactamases and prevents the inactivation of partnered β-lactam antibiotics, FEP's mechanism of action is equivalent to other β-lactams as it inhibits bacterial cell wall biosynthesis via covalent attachment to penicillin-binding proteins (PBPs) and impedes the final transpeptidation step of peptidoglycan synthesis.

Pairing META with FEP restores the activity of this cephalosporin to its original potency and spectrum, as demonstrated in in vitro and in vivo mice models, and extends its activity against Extended-Spectrum Beta-Lactamase (ESBL) producing Enterobacterales [14]. More precisely, META was shown to irreversibly bind to and inactivate Ambler Class A β-lactamases (including SHV-type ESBLs, TEM-type ESBLs, CTX-M-14, CTX-M-15, KPC-2, and KPC-3) [15]. With FEP being stable against AmpC and OXA-48 producers, the FEP/META combination was shown to be stable against Ambler Classes A, C, and D β-lactamases. In addition, in vitro studies demonstrated high susceptibility against OXA-48-producing Enterobacterales, including coproducers of ESBLs or AmpC. Comparatively, the combination was less effective against KPC carbapenemase producers, and META did not show activity against metallo-β-lactamases (NDM, VIM) or added value compared to FEP alone for *P. aeruginosa* and *A. baumannii* [16].

Previous studies have examined FEP’s physicochemical stability under various storage conditions [17,18,19]. Rabouan-Guyon [17] demonstrated that FEP diluted in (0.9% NaCl) or 5% dextrose (D5W) at 8 mg/mL in polyethylene containers was stable up to 48 h at 24 +/− 2 °C in daylight or 15 days at 4 +/− 2 °C protected from light. Stewart [18] has demonstrated that FEP, in polypropylene syringes, was stable for up to 2 days in solutions (sterile water, 0.9% NaCl, and only at 200 ng/mL in D5W) at 22–24 °C. Loeuille [19] concluded that FEP was stable up to 24 h with 0.9% NaCl and D5W in polypropylene syringes, but in elastomeric devices (EDs) at 37 °C, visual modification appears after 6 h.

Given the use of FEP in prolonged infusions and the growing interest in optimizing PK/PD and exposure of new BL/BLI combinations via prolonged-infusion strategies, this study evaluates the physicochemical stability of FEP/META under commonly used conditions. Stability was assessed in 0.9% NaCl and D5W, at multiple concentrations, in polypropylene syringes (unprotected from light) and in elastomeric devices (protected from light), at both refrigerated and elevated temperatures.

Temperature and light exposition parameters studied in these experiments were chosen to be close to the parameters encountered in clinical practice, and solvents were chosen per the Summary of Product Characteristics (SmpC) recommendation [12,13]. Physical stability results were assessed per the European Pharmacopoeia monograph 2.9.19 and 2.9.20 [20,21]. Chemical stability results were assessed per Drug Stability Guidelines, 2008 [22], and European Consensus [23].

To further understand FEP/META stability and its behavior either in 0.9% NaCl and D5W, samples from the stability experiments were analyzed via Liquid Chromatography–Electrospray Ionization–Quadrupole Time-of-Flight–Mass Spectrometry (LC-ESI-QTOF-MS) assays to determine degradation products of FEP and META individually and of the FEP/META combination.

## 2. Results

### 2.1. Chemical Stability of FEP/META by HPLC Assay

#### 2.1.1. Validation of the Method

The retention time of FEP was 5.18 min and 2.14 min for META. Linearity was demonstrated with FEP powder, META powder, and the associated FEP/META, with an R^2^ value in the range of [0.99962; 0.99995]. ANOVA (non-linearity) agreed with the linear model of our method (Fexp < Fth = 3.71) for FEP powder, META powder, and the associated FEP/META. Accuracy was validated with FEP powder [98.11–101.24%], META powder [98.85–101.56%], and the associated FEP/META. The limit of detection (LOD) and limit of quantification (LOQ) were determined as 9.96 µg/mL/30.2 µg/mL for FEP powder, 2.16 µg/mL/6.55 µg/mL for META powder, 14.45 µg/mL/43.79 µg/mL for FEP in association with META, and 1.36 µg/mL/4.12 µg/mL for META in association with FEP. The homogeneity of variances was demonstrated for FEP powder (Cochran’s test: Cexp = 0.536 < Cth(5%; 3;5) = 0.684), for META powder (Cexp = 385 < Cth(5%; 3;5) = 0.684), for FEP in association (Cexp = 0.565 < Cth(5%; 3;5) = 0.684), and for META in association (Cexp = 0.350 < Cth(5%; 3;5) = 0.684). Results for intra-day and inter-day precisions are presented in Table S1 (Supplementary Material). The results obtained for all validation parameters were within the established acceptance criteria, demonstrating that the HPLC method was valid for its intended purpose.

Mass balances of forced degradation for FEP and META are presented in Tables S2 and S3 (Supplementary Material), respectively. A degradation between 10.5% in acid conditions and 20.7% in photolytic conditions was found for FEP, and between 3.9% in photolytic conditions and 24.3% in alkaline conditions for META.

#### 2.1.2. Chemical Stability

**Figure 2:**
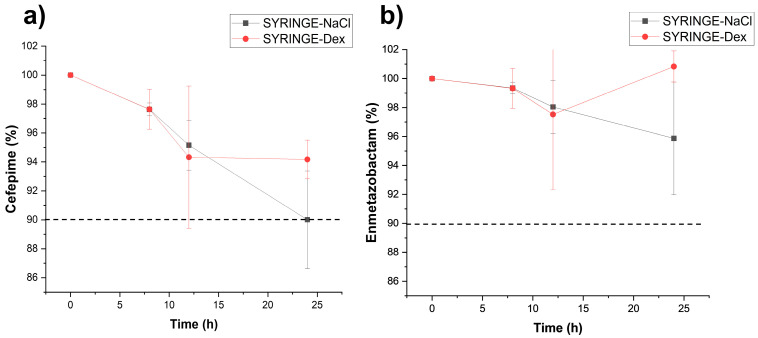
Stability of FEP (**a**) and META (**b**) at 125/31.25 mg/mL when stored in syringes at room temperature using either 0.9% NaCl or D5W as diluents.

**Figure 3:**
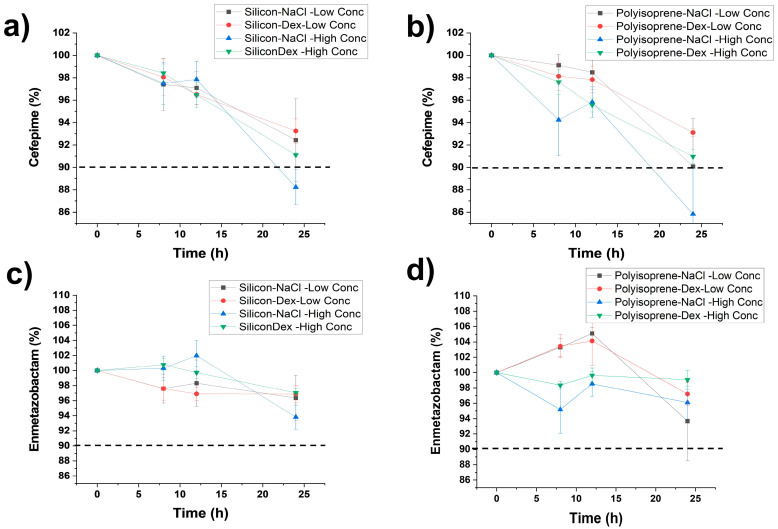
Chemical stability of FEP (**a**,**b**) and META (**c**,**d**) when stored in ED (silicone or polyisoprene) at 32 °C using either 0.9% NaCl or D5W as diluents. Key: High concentration of FEP/META refers to 50/12.5 mg/mL; low concentration of FEP/META refers to 25/6.25 mg/mL, respectively.

The remaining percentages of the initial concentration of FEP and META in association with different concentrations in syringes at room temperature and ED at 32 °C are shown in Figure 2 and Figure 3. Further details are shown in Tables S4 and S5 (Supplementary Material) for FEP and META, respectively. When stored in syringes at 20–25 °C and ED at 32 °C, META retained more than 90% of Ci up to 24 h. FEP retained more than 90% of Ci up to 24 h in D5W, while in 0.9% NaCl, FEP retained more than 90% of Ci up to 12 h (Table 1). After 24 h of storage, two additional peaks were detected. One of them, at 5 min, was attributed to 5-HMF and was only present in samples containing D5W as solvent. The other additional peak detected at 3.2 min was not identified.

#### 2.1.3. Stability of FEP and META at 25/6.25 mg/mL When Stored in Polyisoprene ED at 2–8 °C Using 0.9% NaCl as Diluent

**Figure 4:**
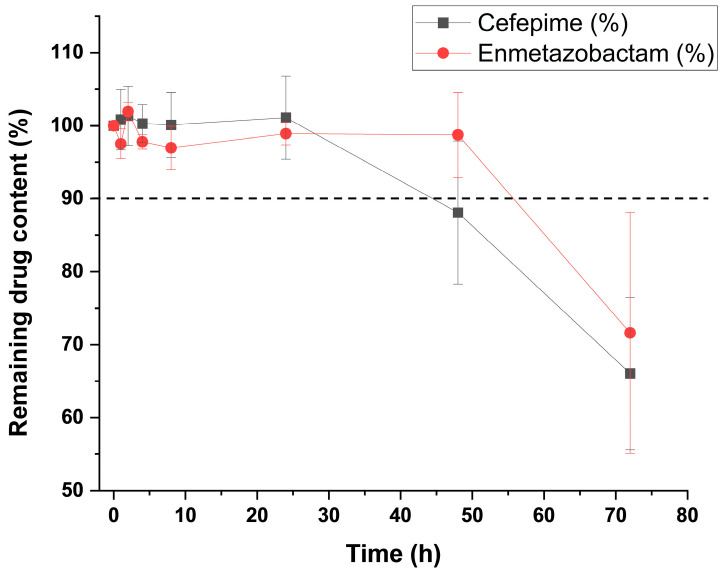
Stability of FEP and META at 25/6.25 mg/mL when stored in polyisoprene ED at 2–8 °C using 0.9% NaCl as diluent.

An LC-QQQ-MS assay was performed to determine the Ci of FEP and META in association diluted in 0.9% NaCl at 25/6.25 mg/mL, stored under refrigerated conditions (2–8 °C) in polyisoprene ED. The LOQ and LOD of the method for META were 0.003 and 0.0008 mg/L, respectively, while the LOQ and LOD for FEP were 0.002 and 0.0005 mg/L, respectively. Results are shown in Figure 4. META retained more than 90% of Ci up to 48 h, while FEP retained more than 90% of Ci up to 24 h in 0.9% NaCl. Further details are described in Table S6 (Supplementary Material).

### 2.2. LC-ESI-QTOF-MS Assay

**Figure 5:**
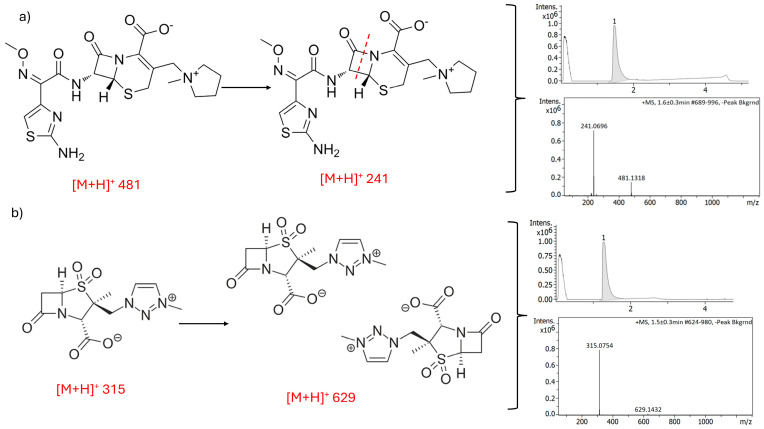
MS/MS spectra and standard chromatogram of FEP (**a**) and META (**b**) raw unprocessed materials using LC-ESI-QTOD-MS.

In Figure 5, the LC-MS mass fragmentation is shown for both FEP and META. In this case, both components were pure and not exposed to degradation before analysis. In the case of FEP (Figure 5a), a main peak was observed in the chromatogram corresponding to a [M + H]^+^ of 481, as well as a fragment of [M + H]^+^ of 241. In the case of META (Figure 5b), a single peak was observed in the chromatogram, corresponding to a mass of [M + H]^+^ 315 and an adduct of 629, possibly due to the strong interaction between two molecules in solution.

### 2.3. Determination of Degradation Product in Solution

**Figure 6:**
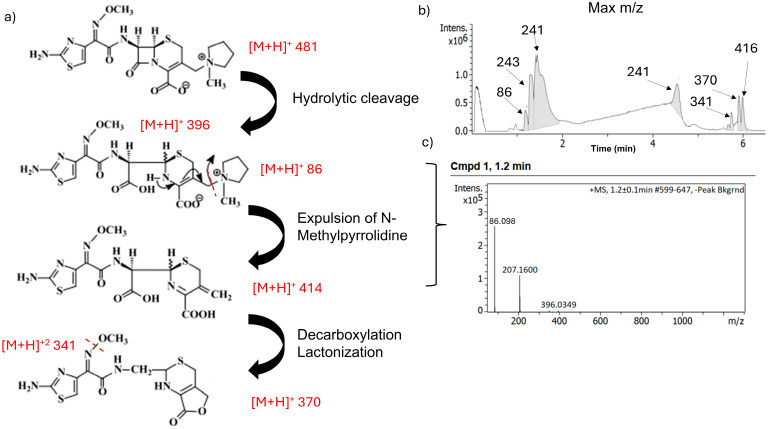
Fragmentation pattern of FEP alone after exposure for 24 h at 32 °C (**a**), standard chromatogram (**b**), and MS/MS spectra of compound 1 at 1.2 min (**c**).

After storage of FEP alone at 32 °C for 24 h in a 0.9% NaCl solution, several degradation products were found (Figure 6).

**Figure 7:**
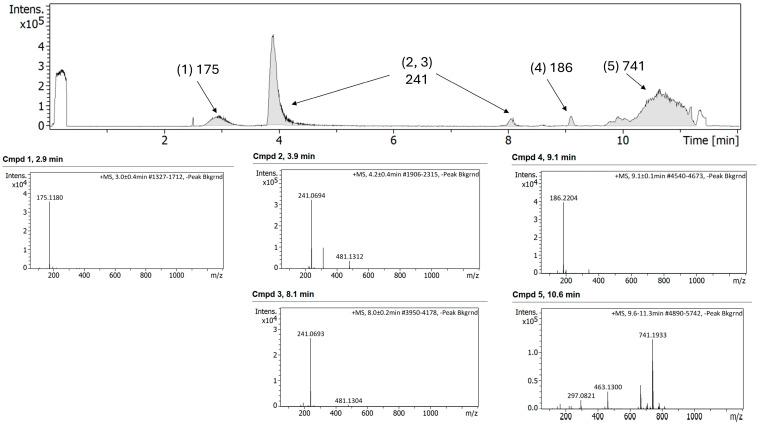
Chromatogram (**top panel**) and MS/MS spectra of different compounds (**bottom panel**) of FEP/META association at 25/6.25 mg/mL, respectively, before exposure to degradation.

The LC–MS profile of FEP alone after exposure for 24 h at 32 °C was significantly different. A total of seven compounds were detected at the following retention times: 1.2, 1.3, 1.4, 4.5, 5.7, 5.7, and 5.9 min, corresponding to mass fragmentation (241 peak) but also to the presence of degradants. It is worth noting the peak of *m*/*z* 86, which corresponds to the N-methylpyrrolidine. FEP undergoes a hydrolytic cleavage that ends with the expulsion of N-methylpyrrolidine (NMP) and afterward triggers the decarboxylation and lactonization of the molecule with an *m*/*z* of 370 (Figure 7) [24].

Interestingly, the LC-MS profile of the combination FEP/META before exposure to any degradation showed significant differences compared to the single components (Figure 7). The [M + H]^+^ of 315 for META was not observed. Instead, a ring cleavage fragment (~C6H8NO3S) with an *m*/*z* of 175 was present in all the samples (peak 1). This peak corresponds to a META fragmentation occurring during Electrospray Ionization (ESI). In the case of the FEP, the mass fragment of 241 was observed (peaks 2 and 3). Additionally, a peak with a *m*/*z* of 741 was present in the sample, which may be an adduct resulting from an electrostatic interaction between FEP and META formed in solution.

**Figure 8:**
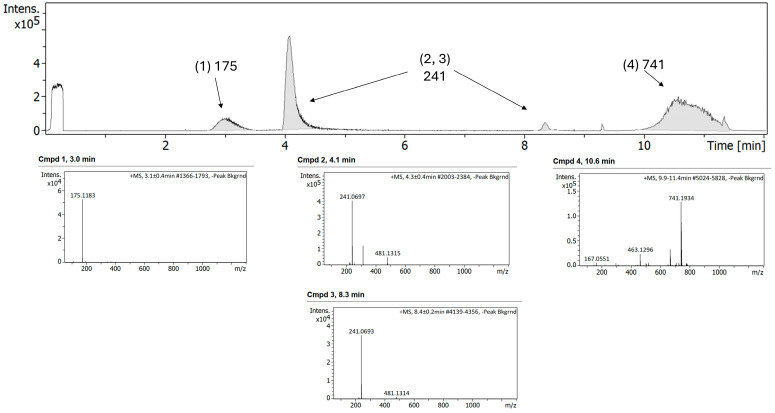
Chromatogram (**top panel**) and MS/MS spectra of different compounds (**bottom panel**) of FEP/META association at 25/6.25 mg/mL, respectively, using 0.9% NaCl diluent and after storage in polyisoprene ED and exposure to 32 °C for 24 h.

After exposure of the FEP/META combination using 0.9% NaCl as diluent and stored at 32 °C in polyisoprene ED for 24 h, degradation peaks observed in the FEP alone were not encountered with the combination FEP/META, which may be related to higher stability over hydrolytic cleavage (Figure 8).

**Figure 9:**
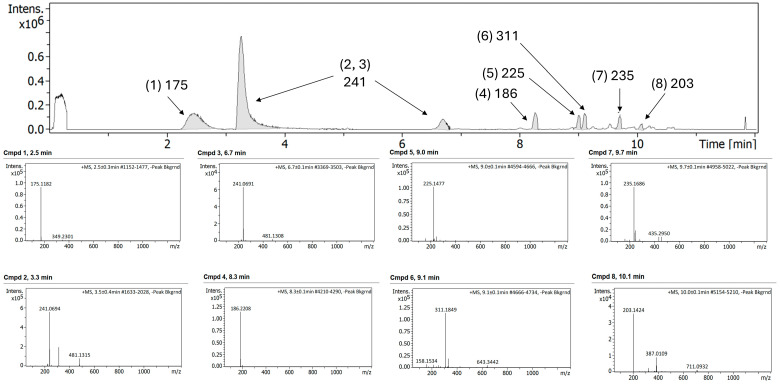
Chromatogram (**top panel**) and MS/MS spectra of different compounds (**bottom panel**) of FEP/META association at 125/31.25 mg/mL, respectively, before exposure to degradation.

In the case of FEP/META stored in polypropylene syringes, a greater number of peaks were observed in the LC–MS chromatogram before exposure to a change in temperature (25 °C) (Figure 9). It is worth noting that the concentration of both components in the syringes was five-fold higher. This can impact the electrostatic interactions formed between FEP and META, as in this case, adducts with a larger molecular weight were not encountered.

**Figure 10:**
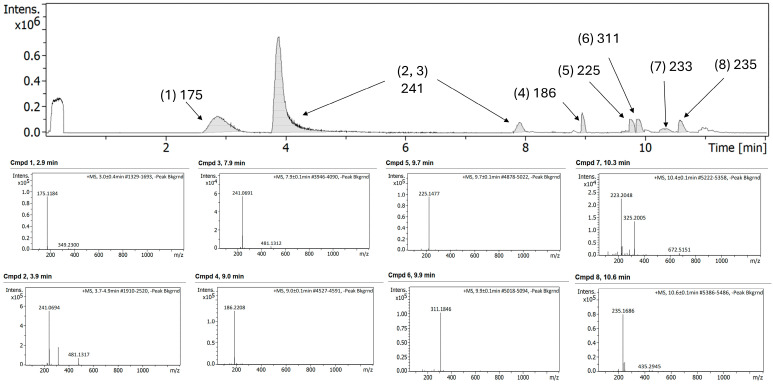
Chromatogram (**top panel**) and MS/MS spectra of different compounds (**bottom panel**) of FEP/META association at 125/31.25 mg/mL, respectively, after storage in syringes and exposure to 25 °C for 24 h.

After exposure to 25 °C over 24 h, similar peaks were observed compared to the undegraded formulation (Figure 10). Peaks related to FEP hydrolytic cleavage were not present.

**Figure 11:**
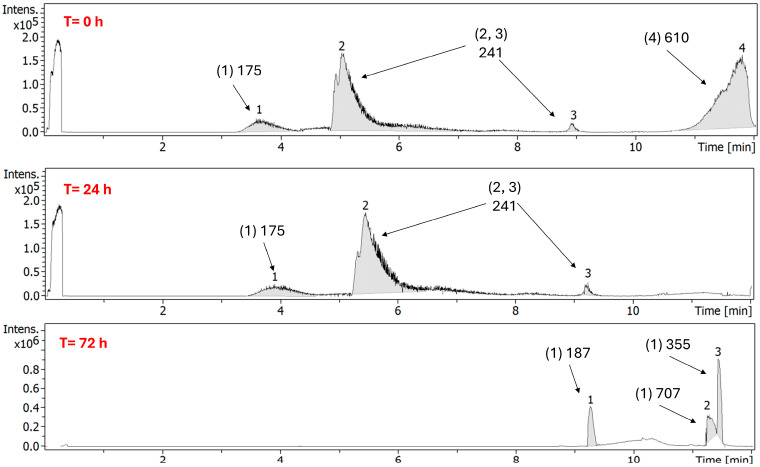
Chromatograms of FEP/META association at 25/6.25 mg/mL at 5 °C using 0.9% NACL as diluent, after storage in polyisoprene ED and exposure to refrigerated conditions (2–8 °C) for 0, 24, and 72 h.

In the case of FEP/META stored in polyisoprene ED at 5 °C using 0.9% NaCl as diluent, it is worth noting the transition from 0 to 24 and 72 h (Figure 11).

At 24 h, both FEP and META showed higher stability than 90% of the Ci at the condition of 2–8 °C in 0.9% NaCl in polyisoprene ED. An adduct peak with an *m*/*z* of 610 was present at that time. At 48 h, META remained stable, but FEP showed degradation below 90% of the Ci. Surprisingly, no adduct was present in solution. After 72 h, the main fragments attributed to META (*m*/*z* of 275) and FEP (*m*/*z* of 241) disappeared, which can be attributed to the extensive degradation found for both drugs, especially FEP.

### 2.4. pH Evaluation

The pH values remained relatively stable at 22–25 °C and 32 °C, with variations of less than one pH unit in all containers and all solvents (Table 2). pH was not evaluated at 5 °C. After 8 h, the greatest change observed was a decrease of 0.09 units; after 12 h, the maximum variation was −0.07. At 24 h, the largest shift was an increase of 0.19 pH units, recorded in the polyisoprene elastomeric device (ED) preparation diluted with 0.9% NaCl, exposed to light, and stored at 32 °C.

### 2.5. Physical Stability

FEP/META physicochemical stability reported in Table 3 was defined by the first parameter that did not meet the predefined acceptance criteria (chemical stability ≥ 90% of the initial concentration, compliant visual inspection, or compliant subvisible particle count for each container–diluent combination). In the case of FEP/META color, yellowing was not taken into account because it is related to FEP and is consistent with its SmPC [8,9,22].

#### 2.5.1. Visual Examination

Visual inspection results at 22–25 °C and 32 °C are detailed in the Supplementary Materials (Figures S1–S5). Visual inspection was not performed at 2–8 °C. For solutions stored in polypropylene syringes, no visible changes were observed during the first 8 h. Initially, the solutions appeared slightly yellow; by 12 h (T12 h), the yellowing became more pronounced, and by 24 h (T24 h), a significant color change had developed. Similar behavior was observed for solutions at 25/6.25 mg/mL, both in silicone and polyisoprene elastomeric devices (EDs), regardless of the diluent used (0.9% NaCl or D5W). At the higher concentration of 50/12.5 mg/mL, slight yellowing was already evident by 8 h (T8 h), with a marked color shift appearing at T12 h. Notably, this discoloration at 12 h was more pronounced than the change seen in the lower-concentration solutions at 24 h (Table 1).

#### 2.5.2. Subvisual Examination

All solutions in polypropylene syringes complied with particle limits at every time point for both solvents. In silicone ED at 25/6.25 mg/mL at 32 °C, all samples (0.9% NaCl and D5W) remained compliant up to 24 h. At the higher concentration of 50/12.5 mg/mL, at 32 °C, compliance was maintained up to 12 h in 0.9% NaCl, and up to 24 h in D5W. In polyisoprene ED at 25/6.25 mg/mL at 32 °C, two out of three samples per solvent met the specifications up to 12 h. For the 50/12.5 mg/mL preparations, all samples remained within acceptable limits up to 12 h (Table 1). Table 3 summarizes the stability duration for each condition.

## 3. Discussion

The present study provides the first comprehensive physicochemical stability evaluation of the fixed-dose combination FEP/META across clinically relevant concentrations, diluents, and infusion systems. Table 3 presents a guidance table providing an overview of the physicochemical stability of FEP/META across different containers, diluents, and storage conditions. Physicochemical stability was defined by the first parameter that failed to meet the predefined acceptance criteria, i.e., for chemical stability ≥ 90% of the initial concentration, acceptable visual inspection, or acceptable subvisible particle counts for each condition.

In our study, we demonstrated that FEP/META solutions in polypropylene syringes at 125/31.25 mg/mL diluted in 0.9% NaCl or D5W were chemically and physically stable for 12 h at 22–25 °C with light exposition, bearing in mind a 90% remaining drug content as a threshold. In silicone ED, at 32 °C, stability was enhanced up to 24 h in D5W at all concentrations tested (25/6.25 mg/mL and 50/12.5 mg/mL), which can be attributed to the formation of a complex in solution between FEP and META. META has significantly greater stability in aqueous solutions than FEP and, due to its zwitterionic behavior, is more likely to interact with FEP compared to other β-lactamases. The chemical stability of the combination was better for both drugs when EDs were stored under refrigerated conditions, for at least 24 h for FEP and 48 h for META in 0.9% NaCl. pH measurements were compliant at 24 h at 22–25 °C and 32 °C in all containers. pH evaluation and visual inspection were not performed at 2–8 °C. Such stability under a variety of settings supports the feasibility of prolonged or continuous infusion regimens, which are increasingly recommended to optimize β-lactam exposure in critically ill patients.

However, one of the limiting factors for their use in OPAT is the yellowish discoloration observed after 12 h at higher temperatures (Figures S1–S5 from Supplementary Material), as this color change was not observed when samples were stored under refrigerated conditions. During the stability studies of FEP/META solutions, a progressive and intense yellow discoloration was observed, particularly under elevated temperatures and prolonged storage. This visual change was more pronounced at higher concentrations and beyond 12 h. Such yellowing is consistent with the known degradation pathways of cefepime, which primarily occur via hydrolysis of the β-lactam ring and oxidative processes in an aqueous solution [19,24]. These degradation pathways can produce chromophoric compounds—such as imine derivatives—that contribute to the yellow-to-brown color shift [25]. The Summary of Product Characteristics (SmPC) for both FEP alone and the FEP/META combination notes that a change in color from yellow to brown may occur after reconstitution [12,13,26]. These documents also clarify that such a color change does not affect the drug’s efficacy, provided storage conditions are respected. Importantly, META is not identified as the source of discoloration, suggesting that FEP is the primary contributor.

Baririan [27] hypothesized that unidentified degradation products might be responsible for the observed color change [23]. One suspected compound is N-methylpyrrolidine (NMP), a known degradation product of FEP. Although NMP is regulated to remain below 0.3% (*v*/*v*) of total volume, its role in color development remains uncertain. According to the Sigma-Aldrich^®^ safety data sheet, NMP is described as a colorless liquid [28], which complicates the hypothesis that it contributes directly to yellowing. This discrepancy suggests that either a transformed derivative of NMP alone or in combination with imine derivatives or another degradation product may be responsible, underscoring the need for further analytical characterization.

Importantly, META itself was not implicated in discoloration and remained chemically stable throughout the observation period. Rather, LC-ESI-QTOF-MS data indicated that META forms a reversible electrostatic complex with FEP in solution, mitigating hydrolytic cleavage and thereby delaying the formation of these degradation products. This interaction could explain the improved stability profile of the FEP/META combination compared with FEP alone.

Clinically, these stability data are highly relevant to current therapeutic paradigms advocating prolonged FEP/META (4-h) or continuous infusions in critically ill patients. In such patients, a shortened β-lactam half-life may result in subtherapeutic exposure during intermittent dosing. The absence of significant degradation during the 4 h infusion period confirms the chemical integrity of the formulation during the approved dose and infusion time for hospital-acquired and ventilator-associated pneumonia (HAP/VAP) and for patients with augmented renal function.

The results also have implications for OPAT. The demonstrated stability of FEP/META indicates that the combination can be a suitable antibiotic option to be used within a range of 12–24 h of continuous infusion. This enables daily home-based administration of FEP/META while preserving both therapeutic efficacy and safety. The potential to administer this combination via portable elastomeric pumps may substantially improve patient quality of life and reduce hospital length of stay. Cold-chain handling (e.g., the use of cooling sleeves during infusion) could mitigate color change and degradation risk.

## 4. Materials and Methods

Water for chromatography was obtained using a reverse-osmosis system (Millipore Iberica, Madrid, Spain), with a resistivity of <15 MΩ cm, and methanol for high-performance liquid chromatography (HPLC) (VWR Chemicals, Ph. Eur. Grade, purity ≥99.8%, Radnor, PA, USA; batch: 22K104007; 23E024010 were purchased from Merck (Madrid, Spain) was used for the mobile phase. Hydrochloric acid 1M (VWR Chemicals; batch: 220510C004), sodium hydroxide 0.1M (VWR Chemicals; batch: 210513C004), and hydrogen peroxide 30% (Supelco, batch: K54376510222) were used for the forced degradation of FEP and META and were purchased from Sigma-Aldrich (Madrid, Spain). 5-hydroxymethylfurfural (5-HMF) (Acros Organics, batch: A0406699), arginine powder (Cooper; batch: 21070123/A), and N-Methylpyrrolidine (batch: 10225988, Thermo Scientific) were used during the validation of the analytical method and the stability study to evaluate additional peaks. Formic acid and LC/MS-grade acetonitrile were purchased from Fisher Scientific (Madrid, Spain). A total of 2 g of FEP powder for injection (PanPharma, batch: T2-10; T2-13), META for intravenous use (Advanz Pharma, Galway, Ireland), and FEP/META (Advanz Pharma, batch: 2003KFQAKA) were used for the validation analytical method. A 0.9% NaCl 500 mL glass vial (Chaix et du Marais, Lavoisier, batch: 3F488), D5W 250 mL glass vial (Chaix et du Marais, Lavoisier, batch: 2F516), and the drug associations were used for the preparation of syringes and EDs. For the preparation of solution tests, drugs were stored in polypropylene syringes (BD Plastipak, batch: 2304711), in polyisoprene ED (Baxter, batch: 22M013), and in silicone ED (ACE Medical, batch: A220420).

### 4.1. Preparation of FEP/META Solutions

#### 4.1.1. 125/31.25 mg/mL in Polypropylene Syringes

Three vials of FEP/META (2 g/0.5 g) were each reconstituted with 10 mL of either 0.9% NaCl or D5W. The reconstituted contents of all three vials were then withdrawn using a 50 mL syringe. Additional diluent (0.9% NaCl or D5W) was added to reach a final volume of 48 mL, achieving a concentration of 125/31.25 mg/mL. Four syringes were prepared per diluent; one syringe from each group was dedicated to pH measurement.

#### 4.1.2. 25/6.25 mg/mL in Elastomeric Devices (EDs)

Three vials of FEP/META (2 g/0.5 g) were reconstituted with 10 mL of 0.9% NaCl or D5W. The combined contents were withdrawn with a 50 mL syringe and transferred into elastomeric devices made of either silicone or polyisoprene. Additional diluent was added to a final volume of 240 mL, resulting in a concentration of 25/6.25 mg/mL. Three devices per diluent were prepared.

#### 4.1.3. 50/12.5 mg/mL in Elastomeric Devices (EDs)

As above, three vials of FEP/META (2 g/0.5 g) were reconstituted with 10 mL of 0.9% NaCl or D5W. The combined solutions were transferred into EDs (silicone or polyisoprene) using a 50 mL syringe. The total volume was increased to 120 mL with the same diluent, yielding a final concentration of 50/12.5 mg/mL. Three devices per diluent were prepared.

### 4.2. Study Design

Syringes were stored at 22–25 °C, exposed to light, while ED at 32 °C, protected from light. Stability was evaluated at different time points, 8, 12, and 24 h, when stored at 22–25 °C and 32 °C, using High Performance Liquid Chromatography (HPLC), pH measurements, and visual and subvisual examinations. FEP-META stored in polyisoprene ED using 0.9% NaCl as diluent was also stored under refrigerated conditions (2–8 °C) and analyzed using LC/MS at different time points (0, 1, 2, 4, 8, 24, 48, and 72 h). At each time, chemical and physical stability were determined. All samples were analyzed in triplicate.

### 4.3. Chemical Stability

#### 4.3.1. HPLC Assay

FEP/META solutions were analyzed via stability-indicating reversed-phase HPLC (RP-HPLC) with photodiode array detection, adapted from a method developed by the manufacturer. The HPLC system consisted of an ELITE LaChrom VWR/Hitachi plus autosampler (Radnor, PA, USA), a VWR photodiode array (PDA) detector L-2455, and a VWR L-2130 HPLC pump. The column used was Phenomenex Kinetex (Torrance, CA, USA), 150 × 4.6 mm, with a particle size of 2.6 μm. The photodiode array detector evaluated the UV spectrum of the chromatographic column effluent every 0.4 s. The wavelength of the analysis spectrum was between 190 and 400 nm. Data acquisition and peak interpretation were performed using EZChrom Elite compact 3.3.2 software (VWR, Agilent Technologies, Madrid, Spain). The mobile phase consisted of water for chromatography (A) and methanol (B) using a gradient method (Table 4). The flow rate was set at 0.9 mL/min, with a volume injection of 2 μL. The detection wavelength was set at 210 nm. The temperature of the column oven is 40 °C.

#### 4.3.2. Validation Method of HPLC Assay

The calibration curve was constructed from the plots of peak area versus concentration obtained from the manufacturer’s powder. The linearity of the method was evaluated with five concentrations (100, 200, 300, 400, and 500 μg/mL) of FEP powder alone and five concentrations (25, 50, 75, 100, and 125 µg/mL) of META powder alone diluted in ultrapure water once a day for three days, and with the association of FEP/META. For the calibration curves, the homogeneity of variances was evaluated using a Cochran test with a significance level set at *p* < 0.05. An analysis of variance (ANOVA) of the linear regression data was performed to assess the significance (*p* < 0.05) of the proposed method. For accuracy, three different solutions of three concentrations were prepared three times a day for 3 days for each molecule. Accuracy was determined as the difference between the mean measured value and the accepted true value. For each molecule, recovery at three concentration levels must be between 98 and 102% to validate accuracy. Values of the limit of detection (LOD) and limit of quantification (LOQ) were determined. The intra-day repeatability was evaluated as recommended by the International Conference on Harmonisation (ICH) Q2 (R1) [29] using three determinations for each concentration at 100, 300, and 500 μg/mL for FEP and 25, 75, and 125 µg/mL for META. For inter-day precision, three determinations for each concentration at 100, 300, and 500 μg/mL for FEP and 25, 75, and 125 µg/mL for META solutions were assayed daily on three different days. Intra-day and inter-day precision was tested for FEP alone, META alone, and FEP in association with META and META in association with FEP.

#### 4.3.3. Forced Degradation and Specificity of HPLC Assay

The stability-indicating capability was evaluated by analyzing forced-degraded FEP and META solutions. This forced degradation aimed to obtain degradation products with retention times different from those of the antibiotic. The objective was to obtain a degradation close to 20% of our molecule of interest [30]. Conditions for degradation were as follows:

- Acidic condition: 1 mL of a 1200 μg/mL FEP solution or 1 mL of a 300 µg/mL META solution were diluted with 1 mL HCl 1 M, stored at room temperature for 1 h, neutralized using 1 mL NaOH 1 M, and diluted with 1 mL ultrapure water to obtain a theoretical concentration of 300 μg/mL and 75 µg/mL (FEP and META, respectively).
- Alkaline condition: 1 mL of a 1200 μg/mL FEP solution or 1 mL of a 300 µg/mL META solution were diluted with 1 mL of NaOH 0.01 M, stored at room temperature for 2 min for FEP and for 5 min for META, neutralized using 1 mL of HCl 0.01 M, and diluted with 1 mL of ultrapure water to obtain a theoretical concentration of 300 μg/mL and 75 µg/mL (FEP and META, respectively).
- Oxidative degradation: 1 mL of a 1200 μg/mL FEP solution or 1 mL of a 300 µg/mL META solution were diluted with 1 mL of H2O2 0.03%, for 2 min for FEP, and with 0.3% H2O2 for 1 min for META and diluted with 3 mL of ultrapure water to obtain a theoretical 300 μg/mL and 75 µg/mL concentration (FEP and META, respectively).
- Heat degradation: 1 mL of a 300 μg/mL FEP solution or 1 mL of a 75 µg/mL META solution was exposed to a temperature of 80 °C for 50 min. Solutions were analyzed directly without dilution.
- Photolytic degradation: 1 mL of a 300 μg/mL FEP solution or 1 mL of a 75 µg/mL META solution was exposed to a UV source of 254 nm for 1800 min for FEP or 1440 min for META. Solutions were analyzed directly without dilution.

The specificity of the method was evaluated by analyzing 0.9% NaCl, D5W, and water for injection solutions. A 100 μg/mL solution of 5-hydroxymethylfurfural (5-HMF), the main degradation product of dextrose, was also analyzed, as it has a maximum absorbance of 284 nm. A solution of arginine at 75 μg/mL was also analyzed, as this is the main excipient found in the manufacturer’s FEP powder.

### 4.4. Sample Dilution for Analysis by RP-HPLC

At each time of analysis, dilutions for HPLC analysis were performed with a target concentration of 300/75 µg/mL, in triplicate for each preparation. Chemical stability was defined as not less than 90% of the initial FEP/META concentration (Ci) and the evolution of potential degradation products [29,30].

### 4.5. Liquid Chromatography–Triple Quadrupole Mass Spectrometry Assay (LC-QQQ-MS)

The stability of FEP/META solutions stored under refrigerated conditions was analyzed using a validated LC-QQQ-MS method [31,32]. The analytic column was a Phenomenex Gemini 5 μm C18 110 Å 150 × 2 mm. Analysis was performed using an LC-MS-8030 Shimadzu (Kyoto, Japan) in a gradient mode. Mobile phase A was composed of H_2_O + 0.1% formic acid, and phase B was composed of acetonitrile. For FEP and META, the gradient mode consisted of 0% phase B from 0–2 min, followed by 2–4 min from 0–95% phase B, 4–5 min at 95% phase B, 5–6 min from 95–0% phase B, and 6–8 min from 95–0% phase B, with a flow rate of 0.4 mL/min and an injection volume of 10 μL. The multiple reaction monitoring (MRM) mode was employed to detect the following quantifier ion transitions: 481.00 > 86.20 (−16eV) and 481.00 > 395.95 (−14 eV) for FEP and 314.60 > 99.00 (−30 eV) and 314.00 > 84.00 (−27 eV) for META. For the preparation of the standards, water was used as a solvent. Identification of degradation products was based on accurate *m*/*z* values, MS/MS fragmentation patterns, and comparison with previously reported data in the literature.

### 4.6. HPLC-QTOF Impact II Assay (LC-ESI-QTOF-MS)

To identify potential reaction intermediates, liquid chromatography coupled with high-resolution mass spectrometry (LC-ESI-QTOF-MS) was employed. An ultra-high performance liquid chromatography (UHPLC) system equipped with a Bruker Elute HPG 1300 pump and interfaced with a QTOF Impact II mass spectrometer (Bruker, Bremen, Germany) was used for analysis. The high-resolution capabilities of the QTOF analyzer enabled the acquisition of exact mass spectra, facilitating the identification of unknown intermediate compounds. Data were acquired in the SCAN(+) mode since the objective was exploratory. Chromatographic separation was achieved using a Bruker Intensity Solo HPLC column (C18–2, 1.8 µm, 2.1 × 100 mm). The mobile phases consisted of water with 0.1% formic acid (Phase A) and acetonitrile (Phase B), delivered at a flow rate of 0.25 mL/min. The gradient program was as follows: 0–1 min at 5% B, 1–6 min linear increase to 95% B, 6–7.5 min at 95% B, 7.5–10 min linear decrease to 5% B, and 10–12 min at 5% B for re-equilibration. The column oven temperature was maintained at 32 °C, and the injection volume was 10 µL. The total run time for each analysis was 12 min.

### 4.7. Sample Preparation and Storage of Supplement Assays Using LC-QQQ-MS and LC-ESI-QTOF-MS

In the first experiment, a total of 25 mg of FEP was accurately weighed and dissolved in 0.5 mL of 0.9% NaCl to achieve a final concentration of 50 mg/mL. A total of 10 mg of META was dissolved in 0.8 mL of 0.9% NaCl to obtain a final concentration of 12.5 mg/mL. For the FEP/META combination, one commercial vial (Exblifep^®^, Mannheim, Germany) containing 2 g of FEP and 0.5 g of META was reconstituted with 10 mL of 0.9% NaCl. The full content of the vial was withdrawn and diluted further with 0.9% NaCl to a final volume of 40 mL, with a concentration of 50/12.5 mg/mL. The resulting solutions were then transferred to amber HPLC glass vials and stored for 24 h at 32 °C in temperature-controlled ovens, protected from light. The LC-ESI-QTOF-MS assay described above was performed (n = 3).

In the second experiment, FEP/META combinations were tested when stored in syringes or polyisoprene EDs. For the high-concentration samples, 60 mL polypropylene syringes were filled with 48 mL of a 125/31.25 mg/mL solution. For the low-concentration samples, polyisoprene elastomeric pumps (Baxter, 10 mL/h, nominal volume of 240 mL) were filled with 240 mL of a 25/6.25 mg/mL solution. Importantly, 0.9% NaCl was used as diluent. The syringes were stored at 25 °C, not protected from light, and the elastomeric pumps at 32 °C in temperature-controlled ovens protected from light for a total duration of 24 h. The LC-ESI-QTOF-MS assay described above was performed (n = 3).

In the third experiment, the FEP/META (25/6.25 mg/mL) combination was tested using LC-QQQ-MS when stored under refrigerated conditions (2–8 °C) in polyisoprene ED for 24 h (n = 3). Importantly, 0.9% NaCl was used as diluent.

### 4.8. pH Measurement

pH was measured at 22–25 °C and 32 °C, for each preparation, and at each time of analysis. pH measurement was performed using a HANNA Edge HI 2002 pH meter (Limena, Italy). pH values were considered to be acceptable if they did not vary by more than 1 pH unit from the initial measurement [30]. pH was not evaluated for solutions stored between 2 and 8 °C due to the limited expected pH drift over short time intervals at low temperatures.

### 4.9. Physical Stability

Physical stability was defined as the absence of particulate formation, haze, color change, and gas evolution. The samples were visually inspected against a white/black background with an unaided eye at each analysis time and for each ED, according to the European Pharmacopoeia monograph 2.9.20. The subvisual aspect was assessed using a particle counter, PAMAS SVSS Germany, to detect microparticles invisible to the naked eye. According to the European Pharmacopoeia monograph 2.9.19, for a container greater than 100 mL (240 mL or 120 mL), the examination is compliant when fewer than 25 particles of 10 µm per mL of solution and fewer than 3 particles of 25 µm per mL of solution are detected. For a container of less than 100 mL, the examination is compliant when fewer than 6000 particles of 10 µm per container and fewer than 600 particles of 25 µm per container are detected [16,17]. Physical stability was not evaluated for solutions stored between 2 and 8 °C.

## 5. Conclusions

Our findings position FEP/META as a chemically stable and clinically feasible candidate for prolonged and continuous infusion therapy in both hospital and home care settings, effectively extending the therapeutic versatility of β-lactam/β-lactamase inhibitor combinations, offering improved probability of pharmacodynamic target attainment and better clinical outcomes, particularly in critically ill patients in intensive care units. 

## Figures and Tables

**Table 1:** Chemical (≥90% of the initial concentration; pH) and physical (visual and subvisual examinations) stability results for FEP/META for each condition studied.

Container	Temperature	Light Exposition	Volume	FEP/META Final Concentration	Solvent	FEP/META Chemical Stability (≥90% of the Ci)	pH (±1 pH Unit)	Visual Examination (Compliant If No Modification Observed)	Subvisual (Compliant If Respects PAMAS Particles Counter per Eur.Pharma. 2.9.19.)
Polypropylene syringe	22–25 °C	Yes	48 mL	125/31.25 mg/mL	0.9% NaCl	12 h	24 h	12 h	24 h
					D5W	24 h	24 h	12 h	24 h
Silicone elastomeric device	32 °C	No	240 mL	25/6.25 mg/mL	0.9% NaCl	12 h	24 h	12 h	24 h
					D5W	24 h	24 h	12 h	24 h
Polyisoprene elastomeric device	32 °C	No	240 mL	25/6.25 mg/mL	0.9% NaCl	12 h	24 h	12 h	12 h
					D5W	24 h	24 h	12 h	12 h
Silicone elastomeric device	32 °C	No	120 mL	50/12.5 mg/mL	0.9% NaCl	12 h	24 h	8 h	12 h
					D5W	24 h	24 h	8 h	24 h
Polyisoprene elastomeric device	32 °C	No	120 mL	50/12.5 mg/mL	0.9% NaCl	12 h	24 h	8 h	12 h
					D5W	24 h	24 h	8 h	12 h
Polyisoprene elastomeric device	2–8 °C	No	240 mL	25/6.25 mg/mL	0.9% NaCl	24 h	not done	not done	not done

**Table 2:** pH evaluation.

			T0	T8 h		T12 h		T24 h	
	Preparation Number	Solvent	pH	pH	≠Relative to T0	pH	≠Relative to T0	pH	≠Relative to T0
Syringe (125/31.25 mg/mL)	1	NaCl 0.9%	4.61	4.54	−0.07	4.58	−0.03	4.54	−0.07
	1	D5W	4.65	4.58	−0.07	4.58	−0.07	4.57	−0.08
Silicone ED (25/6.25 mg/mL)	1	NaCl 0.9%	4.77	4.78	0.01	4.78	0.01	4.97	0.2
	2		4.71	4.74	0.03	4.73	0.02	4.91	0.2
	3		4.63	4.63	0	4.63	0	4.78	0.15
	1	D5W	4.76	4.68	−0.08	4.72	−0.04	4.83	0.07
	2		4.58	4.48	−0.1	4.54	−0.04	4.70	0.12
	3		4.57	4.49	−0.08	4.55	−0.02	4.72	0.15
Silicone ED (50/12.5 mg/mL)	1	NaCl 0.9%	4.53	4.54	0.01	4.58	0.05	4.61	0.08
	2		4.66	4.64	−0.02	4.65	−0.01	4.69	0.03
	3		4.56	4.61	0.05	4.62	0.06	4.71	0.15
	1	D5W	4.65	4.61	−0.04	4.63	−0.02	4.78	0.13
	2		4.58	4.55	−0.03	4.58	0	4.61	0.03
	3		4.65	4.6	−0.05	4.59	−0.06	4.77	0.12
Polyisoprene ED (25/6.25 mg/mL)	1	NaCl 0.9%	4.69	4.6	−0.09	4.64	−0.05	4.78	0.09
	2		4.6	4.56	−0.04	4.56	−0.04	4.78	0.18
	3		4.55	4.53	−0.02	4.55	0	4.70	0.15
	1	D5W	4.52	4.50	−0.02	4.46	−0.06	4.67	0.15
	2		4.48	4.45	−0.03	4.46	−0.02	4.63	0.15
	3		4.49	4.47	−0.02	4.46	−0.03	4.63	0.14
Polyisoprene ED (50/12.5 mg/mL)	1	NaCl 0.9%	4.74	4.70	−0.04	4.74	0	4.90	0.16
	2		4.66	4.63	−0.03	4.64	−0.02	4.84	0.18
	3		4.60	4.60	0	4.59	−0.01	4.79	0.19
	1	D5W	4.74	4.68	−0.06	4.75	0.01	4.94	0.2
	2		4.58	4.56	−0.02	4.59	0.01	4.73	0.15
	3		4.49	4.46	−0.03	4.49	0	4.62	0.13

**Table 3:** Summary of the physicochemical stability of FEP/META in syringes and elastomeric devices under various conditions.

Container	Temperature	Light Exposition	Volume	FEP/META Final Concentration	Solvent	FEP/META Physicochemical Stability
Polypropylene syringe	22–25 °C	Yes	48 mL	125/31.25 mg/mL	0.9% NaCl	12 h
					D5W	24 h
Silicone elastomeric device	32 °C	No	240 mL	25/6.25 mg/mL	0.9% NaCl	12 h
					D5W	24 h
Polyisoprene elastomeric device	32 °C	No	240 mL	25/6.25 mg/mL	0.9% NaCl	12 h
					D5W	12 h
Silicone elastomeric device	32 °C	No	120 mL	50/12.5 mg/mL	0.9% NaCl	12 h
					D5W	24 h
Polyisoprene elastomeric device	32 °C	No	120 mL	50/12.5 mg/mL	0.9% NaCl	12 h
					D5W	12 h
Polyisoprene elastomeric device	2–8 °C	No	240 mL	25/6.25 mg/mL	0.9% NaCl	24 h

**Table 4:** Description of mobile phase gradient.

Time (min)	Water (%)	Methanol (%)
0	95	5
5	40	60
5.1	95	5
12	95	5

## Data Availability

The data that support the findings of this study are available from the corresponding authors, A.C. or E.D., upon reasonable request.

## Supplementary Materials

The following supporting information can be downloaded at: https://www.mdpi.com/article/10.3390/antibiotics15020114/s1, Table S1. Intermediate precision and repeatability assay. Key: SD, standard deviation. Table S2. Mass balance of 300 µg/mL FEP solutions after forced degradation. Table S3. Mass balance of 75 µg/mL META solutions after forced degradation. Table S4. Chemical stability of FEP in association with META. NA, data not available; SD, standard deviation. Table S5. Chemical stability of META in association with FEP. SD, standard deviation. Table S6. Chemical stability of FEP and META in association (at 25/6.25 mg/mL, respectively) when stored under refrigerated conditions (2–8 °C) in polyisoprene ED using NaCl 0.9% as diluent. Figure S1: Visual examination at each time of analysis for FEP/META at 125/31.25 mg/mL in polypropylene syringes in 0.9% NaCl and D5W at 22–25 °C. Figure S2: Visual examination at each time of analysis for FEP/META at 25/6.25 mg/mL in silicone ED in 0.9% NaCl and D5W at 32 °C. Figure S3: Visual examination at each time of analysis for FEP/META at 25/6.25 mg/mL in polyisoprene ED in 0.9% NaCl and D5W at 32 °C. Figure S4: Visual examination at each time of analysis for FEP/META at 50/12.5 mg/mL in silicone ED in 0.9% NaCl and D5W at 32 °C. Figure S5: Visual examination at each time of analysis for FEP/META at 50/12.5 mg/mL in polyisoprene ED in 0.9% NaCl and D5W at 32 °C.
