# Supplementary material for: CEFEPIME/ENMETAZOBACTAM: Physicochemical Stability of a Novel β-Lactam/β-Lactamase Inhibitor Combination in Syringes and Elastomeric Devices
Source: Antibiotics (Basel). 2026 Jan 23;15(2):114. doi: 10.3390/antibiotics15020114 (PMC12937291; doi:10.3390/antibiotics15020114)
Supplement: Supplementary file 1 [file antibiotics-15-00114-s001.zip › antibiotics-4094315-supplementary.pdf]

SUPPLEMENTARY MATERIAL

Table S1. Intermediate precision and repeatability assay. Key: SD, standard deviation.

FEP ALONE			META ALONE			FEP (IN ASSOCIATION)			META (IN ASSOCIATION)		
Concentration	SD	%	Concentration	SD	%	Concentration	SD	%	Concentration	SD	%
100 µg/mL	SD Day 1	0.69%	25 µg/mL	SD Day 1	0.25%	100 µg/mL	SD Day 1	0.79%	25 µg/mL	SD Day 1	0.42%
	SD Day 2	1.01%		SD Day 2	0.45%		SD Day 2	0.87%		SD Day 2	1.66%
	SD Day 3	0.52%		SD Day 3	0.75%		SD Day 3	1.33%		SD Day 3	1.30%
	**SD interday**	**1.27%**		**SD interday**	**1.94%**		**SD interday**	**1.34%**		**SD interday**	**3.77%**
300 µg/mL	SD Day 1	0.65%	75 µg/mL	SD Day 1	0.63%	300 µg/mL	SD Day 1	SD Day 1	75 µg/mL	SD Day 1	1.00%
	SD Day 2	1.65%		SD Day 2	0.87%		SD Day 2	SD Day 2		SD Day 2	0.74%
	SD Day 3	0.56%		SD Day 3	0.72%		SD Day 3	SD Day 3		SD Day 3	0.94%
	**SD interday**	**1.56%**		**SD interday**	**1.92%**		**SD interday**	**1.03%**		**SD interday**	**2.21%**
500 µg/mL	SD Day 1	0.46%	125 µg/mL	SD Day 1	0.55%	500 µg/mL	SD Day 1	SD Day 1	125 µg/mL	SD Day 1	0.55%
	SD Day 2	0.22%		SD Day 2	0.24%		SD Day 2	SD Day 2		SD Day 2	1.12%
	SD Day 3	0.95%		SD Day 3	0.86%		SD Day 3	SD Day 3		SD Day 3	0.07%
	**SD interday**	**1.35%**		**SD interday**	**0.85%**		**SD interday**	**1.07%**		**SD interday**	**2.27%**

Table S2. Mass balance of 300 µg/mL FEP solutions after forced degradation.

				Area				
				**Acid degradation**	**Alkaline degradation**	**Heat degradation**	**Oxidative degradation**	**Photolytic degradation**
**Peaks (n°)**	**Retention times (min)**	**Relative retention**	**Without forced degradation**	HCl 1 M	NaOH 0.01M	80°C	H_2O_2 0.03%	254 nm 1800 min
				60 min	2 min	50 min		
Cefepime	5.173	1	5669060	5071426	4581757	4501671	4834198	4493814
1	1.487	0.287		658950				
2	1.5	0.29	392916		466121	348633	568822	40880
4	1.993	0.385	1163	1492	818	1885		1407
5	2.413	0.466					5168	
6	2.967	0.574					1543	
7	3.193	0.617				73780		
8	3.307	0.639						23799
9	4.167	0.806					24116	
10	4.82	0.932			10997	7603		
11	5.473	1.058		32938	403368	144358		19552
12	5.9	1.141						21006
13	6.007	1.161						20386
14	6.32	1.222		18097				232843
Total mass balance			6063139	5782903	5463061	5077930	5433847	4853687
Degradation (%)				10.5	19.2	20.6	14.7	20.7
Cefepime (%)				89.5	80.8	79.4	85.3	79.3

Table S3. Mass balance of 75 µg/mL META solutions after forced degradation.

				Area				
				**Acid degradation**	**Alkaline degradation**	**Heat degradation**	**Oxidative degradation**	**Photolytic degradation**
**Peaks (n°)**	**Retention times (min)**	**Relative retention**	**Without forced degradation**	HCl 1 M	NaOH 0.01M	80°C	H_2O_2 0.3%	254 nm 1440 min
				60 min	5 min	50 min		
META	2.127	1	894579	775369	677430	727511	802086	859254
1	1.44	0.677			104741			
2	1.467	0.69		273917				
3	1.54	0.724					5502995	
4	1.547	0.727		165016				
5	1.553	0.73			218905	83470		
6	1.56	0.733	1204					35693
7	1.687	0.793	1442					
8	2.467	1.16						9678
9	2.86	1.345				112632		
10	2.873	1.351					3655	
Total mass balance			897225	1214302	1001076	923613	6308736	904625
Degradation (%)				13.3	24.3	18.7	10.3	3.9
Cefepime (%)				86.7	75.7	81.3	89.7	96.1

Table S4. Chemical stability of FEP in association with META. NA, data not available; SD, standard deviation.

			T0h			T8h		T12h		T24h	
	Preparation number	Solvent	Measured conc. (mg/mL)	% relative to initial concentration (Ci)	SD (%)	% relative to Ci	SD (%)	% relative to Ci	SD (%)	% relative to Ci	SD (%)
Syringe (125 mg/mL) 22-25 ºC	1	NaCl 0.9%	131.96	100.00	0.88	97.12	1.34	96.97	1.64	89.05	5.42
	2		132.99	100.00	0.39	97.93	0.63	93.55	0.57	87.23	1.68
	3		131.74	100.00	0.27	97.85	0.57	94.95	0.42	93.75	0.68
	1	D5W	130.06	100.00	0.49	97.34	2.09	97.26	1.03	94.61	0.65
	2		130.37	100.00	1.6	99.15	2.14	97.07	0.39	95.24	1.58
	3		133.66	100.00	0.22	96.44	1.82	*NA*		92.67	0.60
Silicone (25 mg/mL) 32 ºC	1	NaCl 0.9%	25.86	100.00	1.40	96.09	0.62	98.75	0.67	88.16	6.96
	2		26.35	100.00	0.17	96.00	1.82	96.38	1.14	94.63	0.15
	3		26.33	100.00	0.48	100.10	0.76	96.79	1.42	94.50	0.22
	1	D5W	26.76	100.00	0.78	96.92	1.34	96.12	0.83	92.06	2.01
	2		26.39	100.00	0.63	97.29	2.02	95.85	1.06	94.18	1.60
	3		26.07	100.00	1.18	99.95	1.30	97.53	0.44	93.50	2.34
Silicone (50 mg/mL) 32 ºC	1	NaCl 0.9%	51.58	100.00	0.40	96.95	0.89	99.72	0.79	89.12	6.65
	2		52.75	100.00	1.13	95.97	7.14	96.99	1.44	86.44	1.34
	3		53.09	100.00	0.42	99.58	1.27	96.84	2.05	89.14	2.30
	1	D5W	53.15	100.00	0.67	98.55	1.06	96.43	2.27	91.20	1.14
	2		53.19	100.00	0.89	97.54	0.58	95.36	2.25	90.89	0.56
	3		52.44	100.00	0.62	99.14	0.21	97.61	2.55	91.22	0.51
Polyisoprene (25 mg/mL) 32 ºC	1	NaCl 0.9%	26.10	100.00	2.73	97.98	0.58	100.12	1.72	85.94	1.29
	2		26.28	100.00	1.17	99.73	0.39	98.20	1.52	89.79	2.47
	3		27.00	100.00	0.51	99.61	0.94	97.11	2.18	94.51	0.42
	1	D5W	26.53	100.00	1.60	99.46	1.62	99.15	0.08	93.46	1.17
	2		26.92	100.00	1.35	97.07	1.20	96.82	2.38	92.87	0.83
	3		26.81	100.00	1.28	97.91	1.48	97.53	1.64	92.95	1.12
Polyisoprene (50 mg/mL) 32 ºC	1	NaCl 0.9%	52.61	100.00	1.37	92.44	6.75	97.15	0.50	81.38	1.39
	2		53.65	100.00	1.58	92.33	1.51	94.43	1.88	86.46	5.44
	3		53.92	100.00	1.83	97.94	2.72	95.92	1.93	89.36	1.98
	1	D5W	53.59	100.00	1.06	96.64	2.13	94.85	0.83	90.24	2.87
	2		52.84	100.00	1.41	97.34	1.83	95.89	1.58	91.17	1.63
	3		53.22	100.00	2.12	98.86	1.92	95.95	1.92	91.48	1.20

Table S5. Chemical stability of META in association with FEP. SD, standard deviation.

			T0h			T8h		T12h		T24h	
	Preparation number	Solvent	Measured conc. (mg/mL)	% relative to initial concentration (Ci)	SD (%)	% relative to Ci	SD (%)	% relative to Ci	SD (%)	% relative to Ci	SD (%)
Syringe (31.25 mg/mL) 22-25 ºC	1	NaCl 0.9%	30.63	100.00	0.38	98.90	1.60	99.81	1.66	94.41	5.35
	2		30.68	100.00	0.31	99.54	0.31	96.17	0.90	92.92	1.69
	3		30.25	100.00	0.23	99.59	0.37	98.14	0.22	100.28	0.72
	1	D5W	30.02	100.00	0.41	98.87	2.21	100.67	0.99	101.49	0.73
	2		30.20	100.00	1.66	100.87	2.26	100.38	0.14	101.42	1.70
	3		30.67	100.00	0.46	98.24	1.72	91.51	1.99	99.59	0.83
Silicone (6.25 mg/mL) 32 ºC	1	NaCl 0.9%	6.06	100.00	0.45	97.95	0.69	101.86	0.37	92.94	6.79
	2		6.26	100.00	0.06	95.55	1.77	96.26	1.19	98.72	0.18
	3		6.24	100.00	0.56	99.35	0.74	96.86	1.31	97.39	0.92
	1	D5W	6.39	100.00	0.76	96.59	1.42	96.51	0.90	95.61	2.02
	2		6.23	100.00	0.73	96.85	1.91	96.27	1.07	97.90	1.67
	3		6.15	100.00	1.05	99.36	1.30	97.91	0.58	97.11	2.75
Silicone (12.5 mg/mL) 32 ºC	1	NaCl 0.9%	12.03	100.00	0.97	99.82	1.03	104.25	1.05	94.95	7.03
	2		12.32	100.00	1.35	98.98	7.45	101.34	1.49	91.99	1.51
	3		12.57	100.00	0.43	102.11	1.50	100.34	2.00	94.45	2.17
	1	D5W	12.52	100.00	0.50	101.35	0.99	100.57	2.28	96.63	1.16
	2		12.58	100.00	1.23	99.85	0.82	99.06	2.38	96.78	0.50
	3		12.33	100.00	0.50	101.12	0.35	99.51	2.48	97.74	0.75
Polyisoprene (6.25 mg/mL) 32 ºC	1	NaCl 0.9%	6.11	100.00	3.17	101.94	0.58	105.73	1.85	88.42	1.24
	2		6.10	100.00	1.24	103.97	2.14	105.33	1.65	93.91	2.68
	3		6.14	100.00	0.56	103.97	0.39	104.23	2.26	98.67	0.53
	1	D5W	6.13	100.00	1.63	103.97	1.46	107.39	0.02	98.72	1.17
	2		6.35	100.00	2.88	104.63	1.11	103.98	2.28	97.09	0.58
	3		6.31	100.00	1.24	101.73	2.26	101.04	2.25	95.81	1.38
Polyisoprene (12.5 mg/mL) 32 ºC	1	NaCl 0.9%	12.33	100.00	1.40	98.77	7.17	99.1	0.58	87.76	1.63
	2		12.54	100.00	1.61	93.40	1.73	96.72	2.92	93.65	5.63
	3		12.68	100.00	1.88	93.40	2.99	99.81	2.00	96.93	2.07
	1	D5W	12.71	100.00	0.30	97.81	2.12	98.90	0.95	97.72	4.58
	2		12.23	100.00	1.36	98.81	1.85	100.72	1.60	100.14	1.76
	3		12.44	100.00	2.20	98.47	2.00	99.29	1.84	99.35	1.16

Table S6. Chemical stability of FEP and META in association (at 25/6.25 mg/ml, respectively) when stored under refrigerated conditions (2-8 ºC) in polyisoprene ED using NaCl 0.9% as diluent.

Results are expressed as percentage relative to the initial concentration ± standard deviation. Results are the average of three measurements.

Drug	T0h	T1h	T2h	T4h	T8h	T24h	T48h	T72h
	Ci ± SD (%)	Ci ± SD (%)	Ci ± SD (%)	Ci ± SD (%)	Ci ± SD (%)	Ci ± SD (%)	Ci ± SD (%)	Ci ± SD (%)
FEP	100	100.8 ± 4.12	101.3 ± 4.0	100.3 ± 2.6	100.1 ± 4.4	101.1 ± 5.7	88.1 ± 9.8	66.0 ± 10.4
META	100	97.5 ± 2.1	101.9 ± 1.3	97.8 ± 0.9	97.0 ± 2.9	98.9 ± 1.6	97.6 ± 2.5	71.6 ± 16.5

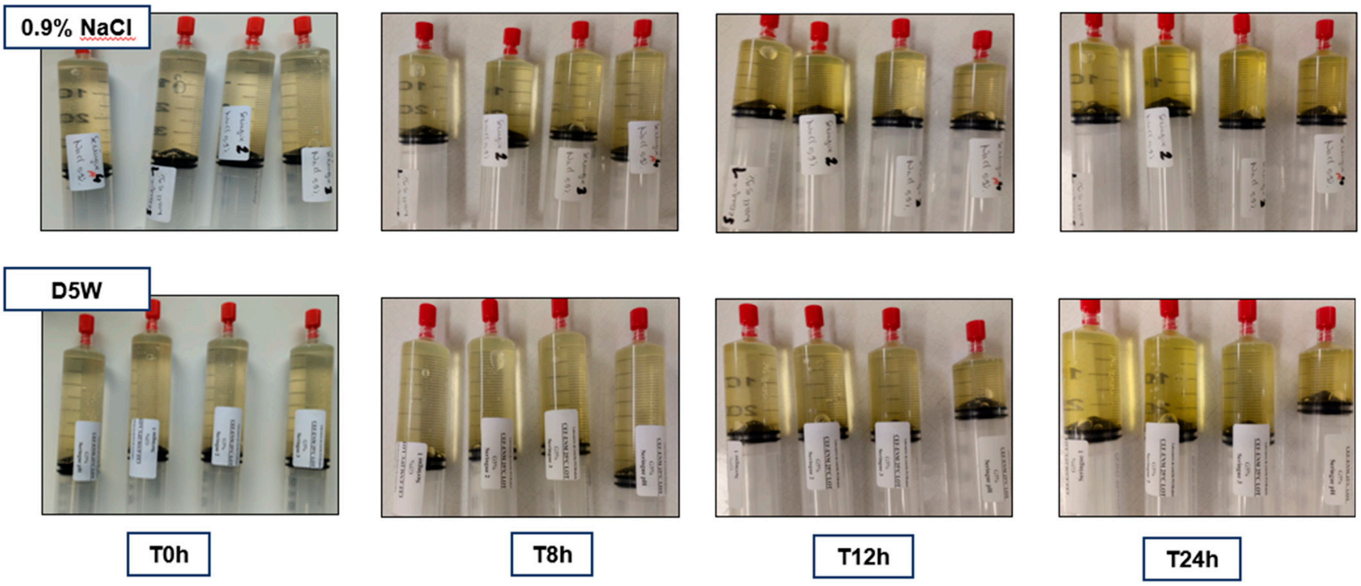

Figure S1: Visual examination at each time of analysis for FEP/META at 125/31.25 mg/mL in polypropylene syringes in 0.9% NaCl and D5W at 22-25 ºC.

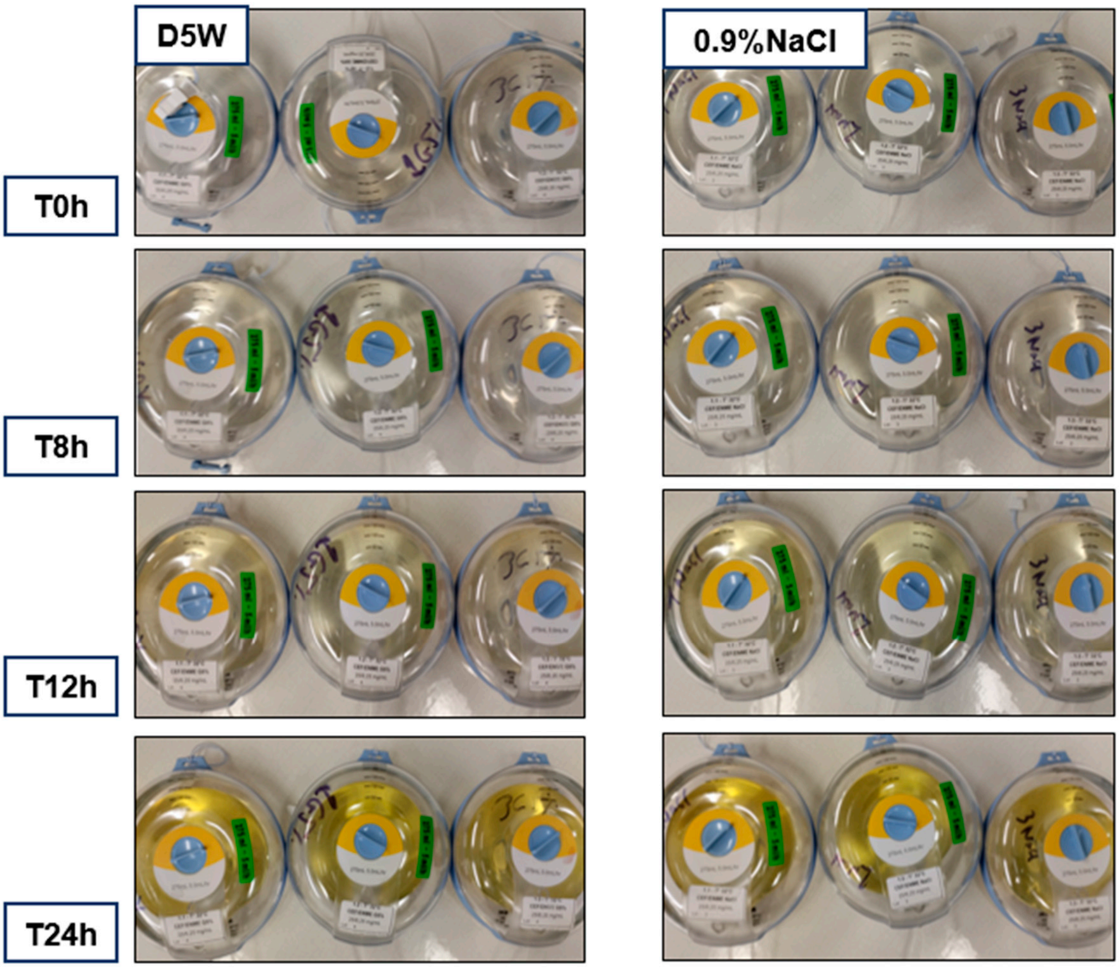

Figure S2: Visual examination at each time of analysis for FEP/META at 25/6.25 mg/mL in silicone ED in 0.9% NaCl and D5W at 32 ºC.

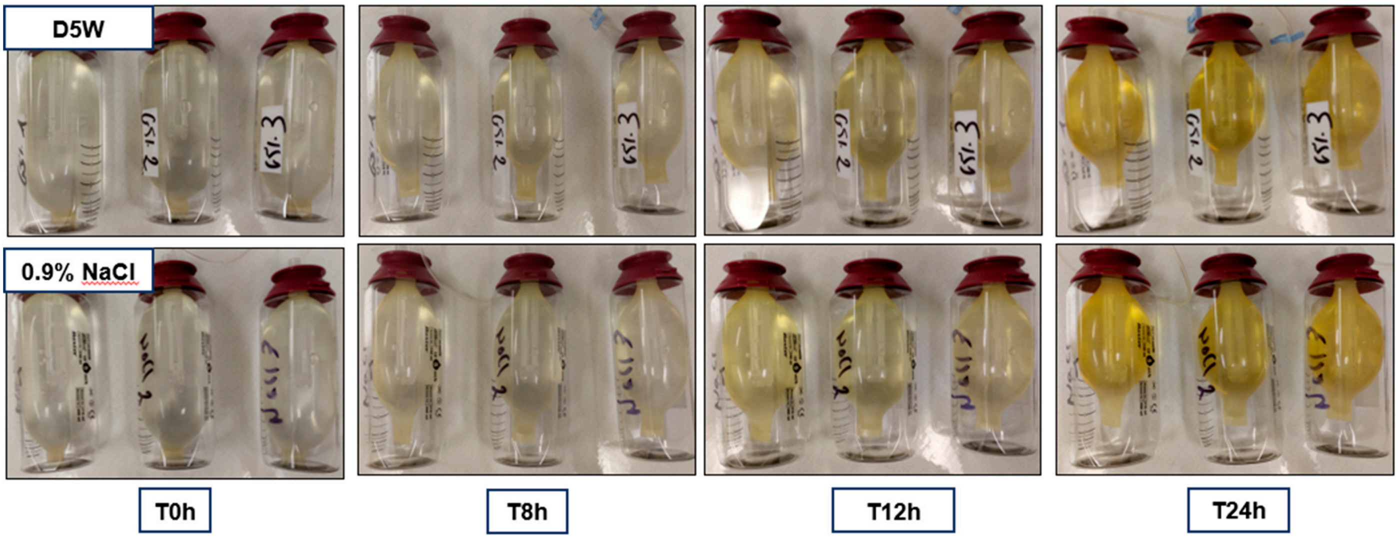

Figure S3: Visual examination at each time of analysis for FEP/META at 25/6.25 mg/mL in polyisoprene ED in 0.9% NaCl and D5W at 32 ºC.

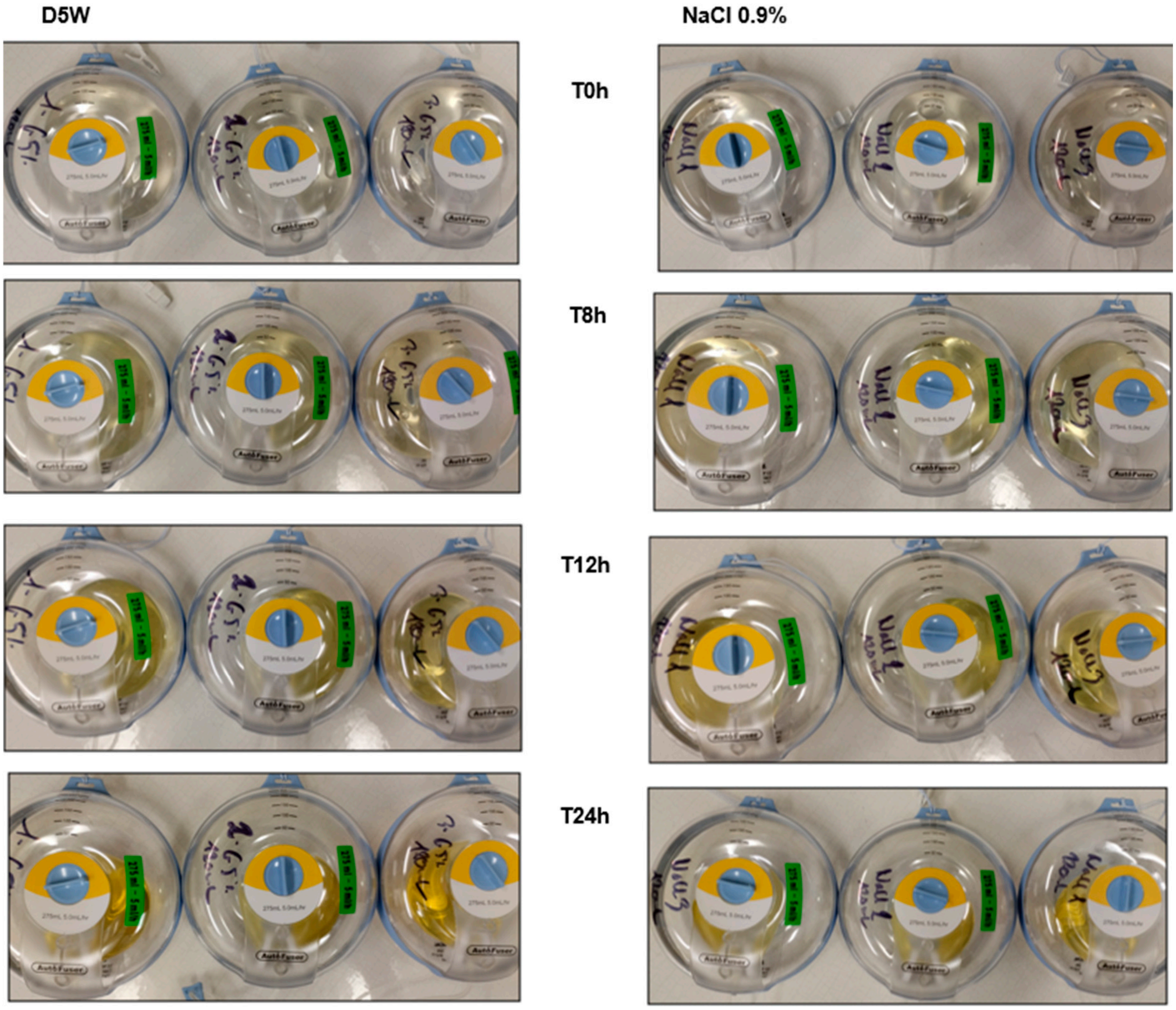

Figure S4: Visual examination at each time of analysis for FEP/META at 50/12.5 mg/mL in silicone ED in 0.9% NaCl and D5W at 32 ºC.

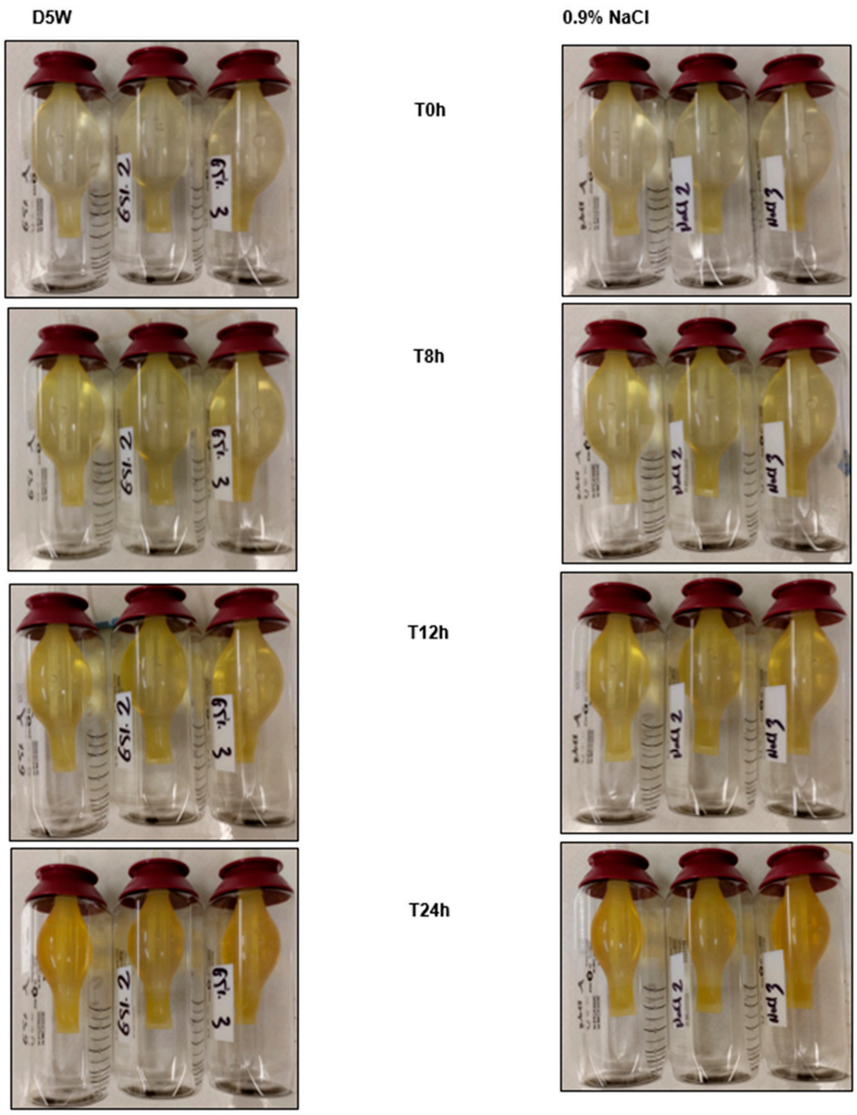

Figure S5: Visual examination at each time of analysis for FEP/META at 50/12.5 mg/mL in polyisoprene ED in 0.9% NaCl and D5W at 32 ºC.
